# Shooting Stars

**Published:** 1883-08

**Authors:** 


### SHOOTING STARS.

An observer carefully watching the heavens on a cloudless night will often see an object that looks like a star, shooting through a short space in the sky, and then suddenly disappearing.

Several of these seeming stars may be seen in the course of an hour. Sometimes they are very faint, and are visible only for a second; sometimes they move more slowly and are seen for several seconds. Sometimes they leave behind a long train of silver light; and sometimes they are so large and brilliant as to illumine the heavens like the bursting of a sky-rocket.

These little bodies are called shooting stars or meteoroids, and the more brilliant ones are known as meteors. Celestial space is full of them. Their number is countless, and they move round the sun in every possible kind of orbit, although as yet nothing is certainly known concerning their origin and constitution.

The earth is constantly encountering the tiny atoms, while making her annual revolution round the sun. Moving in her orbit with a velocity of eighteen miles a second, she meets swarms of meteoroids, usually passing in an opposite direction, with a velocity often as great as twenty-six miles a second.

As the meteoroids plunge headlong against our atmosphere, a tremendous concussion takes place, and so much heat is evolved that the intruders are actually burned up, and the shooting stars are simply the light of the conflagration.

So numerous are the meteoroids that it is supposed that seven millions fall to the earth every twenty-four hours.

The shooting stars we see on clear nights move in every direction, and seem to be scattered without plan or purpose. The case is different with those that cause the meteoric showers.

These move in immense meteor-zones or hoops, the points nearest the sun resting on the earth's orbit, and the point farthest from the sun extending to or beyond the most distant planets.

The earth will pass through one of these zones on the 13th of November and, during the passage, a moderate display of shooting stars may be looked for.

Once in about thirty-three years, she encounters a wonderfully brilliant and concentrated portion of the zone. The meteors then shoot forth in all directions, and in such numbers that the heavens seem to be on fire. A grand display of November meteors is not expected until 1899. The meteors are also known as Leonids.

